# Interest of Chest CT to Assess the Prognosis of SARS-CoV-2 Pneumonia: An In-Hospital-Based Experience in Sub-Saharan Africa

**DOI:** 10.1155/2024/5520174

**Published:** 2024-04-25

**Authors:** Serge Emmanuel Obe -A- Ndzem Holenn, Tacite Kpanya Mazoba, Désiré Yaya Mukanga, Tyna Bongosepe Zokere, Djo Lungela, Jean-Robert Makulo, Steve Ahuka, Angèle Tanzia Mbongo, Antoine Aundu Molua

**Affiliations:** ^1^Department of Radiology and Medical Imaging, Hôpital Médecins de nuit SARL, Kinshasa, Democratic Republic of the Congo; ^2^Department of Radiology and Medical Imaging, Cliniques Universitaires de Kinshasa, Kinshasa, Democratic Republic of the Congo; ^3^Intensive Care Unit, Cliniques Universitaires de Kinshasa, Kinshasa, Democratic Republic of the Congo; ^4^Interdisciplinary Center for Research in Medical Imaging (CIRIMED), University of Kinshasa, Kinshasa, Democratic Republic of the Congo; ^5^Intensive Care Unit, Hôpital Médecins de nuit SARL, Kinshasa, Democratic Republic of the Congo; ^6^COVID-19 Treatment Center, Cliniques Universitaires de Kinshasa, Kinshasa, Democratic Republic of the Congo; ^7^Department of Microbiology, Cliniques Universitaires de Kinshasa, Kinshasa, Democratic Republic of the Congo

## Abstract

**Methods:**

We included all patients with respiratory symptoms (dyspnea, fever, and cough) and/or respiratory failure admitted to the SOS Médecins de nuit SARL hospital, DR Congo, during the 2nd and 3rd waves of the COVID-19 pandemic. The diagnosis of COVID-19 was established based on RT-PCR anti-SARS-CoV-2 tests (G1 (RT-PCR positive) vs. G2 (RT-PCR negative)), and all patients had a chest CT on the day of admission. We retrieved the digital files of patients, precisely the clinical, biological, and chest CT parameters of the day of admission as well as the vital outcome (survival or death). Chest CT were read by a very high-definition console using Advantage Windows software and exported to the hospital network using the RadiAnt DICOM viewer. To determine the threshold for the percentage of lung lesions associated with all-cause mortality, we used ROC curves. Factors associated with death, including chest CT parameters, were investigated using logistic regression analysis.

**Results:**

The study included 200 patients (average age 56.2 ± 15.2 years; 19% diabetics and 4.5% obese), and COVID-19 was confirmed among 56% of them (G1). Chest CT showed that ground glass (72.3 vs. 39.8%), crazy paving (69.6 vs. 17.0%), and consolidation (83.9 vs. 22.7%), with bilateral and peripheral locations (68.8 vs. 30.7%), were more frequent in G1 vs. G2 (*p* < 0.001). No case of pulmonary embolism and fibrosis had been documented. The lung lesions affecting 30% of the parenchyma were informative in predicting death (area under the ROC curve at 0.705, *p* = 0.017). In multivariate analysis, a percentage of lesions affecting 50% of the lung parenchyma increased the risk of dying by 7.194 (1.656-31.250).

**Conclusion:**

The chest CT demonstrated certain characteristic lesions more frequently in patients in whom the diagnosis of COVID-19 was confirmed. The extent of lesions affecting at least half of the lung parenchyma from the first day of admission to hospital increases the risk of death by a factor of 7.

## 1. Introduction

At the beginning of the COVID-19 pandemic, reverse transcriptase polymerase chain reaction (RT-PCR) equipment was not available in several countries of the sub-Saharan African (SSA) region. Given this scarcity, several countries were not able to detect the disease on a large scale in the population [1–3]. Despite the use of SARS-CoV-2 antibodies such as IgM and IgG serological tests, many diagnoses have not been made due to the diagnostic window of these tests which is very wide [4, 5].

To remedy this weakness, in symptomatic patients suspected of having COVID-19, the WHO suggested using chest imaging for the diagnosis of COVID-19 when virological tests are not available; virological tests are available, but the results are slow; and initial virological tests are negative, but a strong clinical suspicion of COVID-19 remains [6, 7]. Indeed radiologists have described some lung lesion characteristics of COVID-19 such as ground-glass opacities and crazy paving appearing in peripheral areas of the lung parenchyma [6–9]. Several publications also report that the combination of the extension of these lesions and the association with images of pulmonary consolidation are correlated with a poor prognosis of the disease [9, 10]. Repeat chest CT are indicated if complications are suspected such as pulmonary embolism. Centrilobular nodules, isolated lobar consolidation, lymphadenopathy, and large pleural effusions are rarely described in COVID-19 patients. Therefore, these lesions suggest other diagnoses or complications.

In Africa, DR Congo in particular, only a few hospitals used chest CT in the diagnosis and the management of patients suspected of COVID-19. They mainly reported bilateral peripheral ground-glass opacities in 60-69% of patients, crazy paving in 19-67%, and consolidation lesions in 58-80% [11–13]. These studies had small sample sizes. One study had examined the chest CT score based on the percentage of lung lesions but failed to establish a link with mortality [14]. The objectives of the present study, carried out during the COVID-19 pandemic in Kinshasa, were to describe chest CT lesions on the first day of admission in patients hospitalized for suspected COVD-19, to determine their association with SARS-CoV-2 infection, and to identify the chest CT descriptions which were associated with death.

## 2. Methods

Our study was carried out from January 1, 2021, to December 31, 2021, during the 2nd and 3rd waves of the COVID-19 pandemic in Kinshasa. We included all patients presenting respiratory symptoms (dyspnea, fever, and cough) and/or biologically confirmed respiratory failure and hospitalized at the Médecins de nuit SARL, a private local hospital of the capital city of Kinshasa in DR Congo. Only patients with a chest CT examination performed during the first 24 hours of admission were included in this study. The SARS-CoV-2 RT-PCR examinations were carried out by the staff of the national laboratory (INRB) using nasal and throat swabs. A negative result was defined by two consecutive negative tests.

Two groups were compared based on the SARS-CoV-2 RT-PCR results: group 1 (patients with at least one positive test) and group 2 (patients with two consecutive negative tests). Other parameters of interest were patient age, sex, SaO2 at admission, comorbidities, chest and lung lesions (type of lesion, location, and percentage of lung parenchyma affected), and the vital issue.

All patients in the study were treated according to national guidelines for the fight against COVID-19 and according to the complications observed [15]. The chest CT examinations were carried out at the Médecins de nuit SARL hospital using a General Electric REVOLUTION 64/128 slice device.

### 2.1. Acquisition Protocol and Chest CT Data Collection Technique

The chest CT sections were obtained with a millimetric helical acquisition on patients in the supine position with the arms raised above the head. The acquisition was made from the pulmonary apex to the upper level of the abdomen in thin sections of 0.625 mm followed by multiplanar reconstruction, MIP, and Mini MIP. The acquisition time for the 0.625 mm × 30 cm sections was achieved in less than 2 seconds. The exams were read on a very high-definition console using Advantage Windows software and exported in the hospital network using RadiAnt DICOM viewer. The reports were produced directly on our RIS LOG software.

In case of suspicion of pulmonary embolism, a CT scan was carried out after an IV injection of the contrast product (1-2 ml/kg with a flow rate of 3-5 ml/second) on a NEMOTO electrical syringe.

### 2.2. Operational Definitions

**Figure 1:**
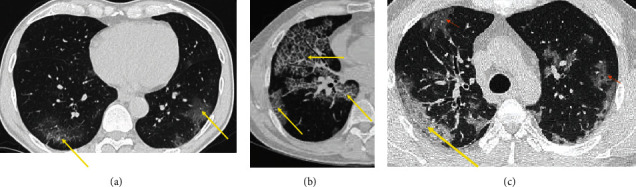
Chest CT lesions: (a) ground glass, (b) crazy paving, and (c) consolidation.

Chest CT lesions were defined as follows: *ground-glass overdensities* (an increase in the density of the lung parenchyma not obliterating the vessels which may be nodular, banded, or patchy with or without) (Figure 1(a)), *crazy paving* (presence of perilobular and/or intralobular reticular opacities within ground-glass overdensities) (Figure 1(b)), and *consolidation* (an homogeneous increase in lung density that erases the vessels and bronchial walls, with persistence of aeric bronchogram) (Figure 1(c)) [16]. The extent of lung lesions was presented in percentages in accordance with the European Society of Radiology (ESR) and the European Society of Thoracic Imaging (ESTI): minimal/absent less than 10%, moderate between 10 and 25%, extensive between 25 and 50%, severe between 50 and 75%, and critical above 75% [9].

### 2.3. Statistical Analysis

Data were exported to IBM SPSS 21 (Statistical Package for Social Sciences), version 21.0, for processing and analyses. Results are presented as means and standard deviation for Gaussian-distributed quantitative data and as relative proportions (%) and absolute frequencies (*n*) for categorical data. The Pearson chi-square test was performed for the comparison of proportions between categorical variables, while Student's *T* test was used to compare quantitative variables. The sensitivity and specificity of the extent of damaged lungs in predicting death was studied using receiver operating characteristic (ROC) curves. The determinants associated with death were sought using binary logistic regression using the step-by-step descending method. The statistical significance threshold (*p* value) was *p* < 0.05.

### 2.4. Ethical Considerations

This study was carried out in strict compliance with the recommendations of the Declaration of Helsinki III. The processing and analysis of the data took place anonymously while respecting confidentiality.

## 3. Results

### 3.1. General Characteristics of Patients

During the study period, 200 patients were hospitalized for SARS-CoV-2 pneumonia. In 112 patients (56%), SARS-CoV-2 infection was confirmed, while in 88 patients (44%), RT-PCR results were negative.  Table 1 shows that the patients had a mean age of 56.2 ± 15.2 years; 19% were diabetic and 4.5% obese, without a statistically significant difference between the two groups. The history of hypertension was 51.8% in patients with confirmed SARS-CoV-2 infection versus 12.5% in the other group (*p* < 0.001).

### 3.2. Chest CT Lesions in Patients

Table 2 which describes the chest CT lesions shows that in the group with confirmed SARS-CoV-2 infection, patients more frequently had ground-glass lesions (72.3%), crazy paving (69.6%), and condensation (83.9%), with bilateral locations in 68.8% of patients. Lesions affecting more than 50% of the lung parenchyma affected 18.8% of COVID-19 patients.

13 patients presented pseudonodular lesions including 9 in G1 and 4 in G2 without statistically significant difference (*p* = 0.320). No case of pulmonary embolism had been documented.

### 3.3. ROC Curve

**Figure 2:**
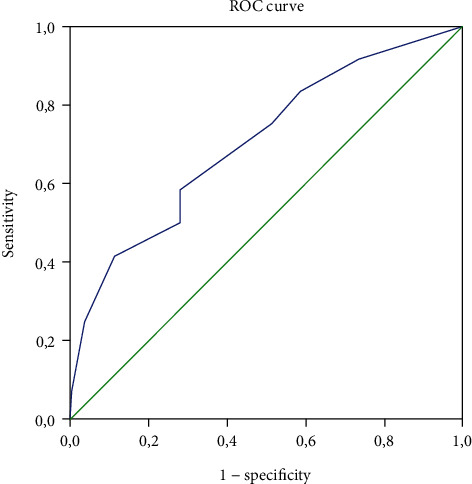
ROC curve sensitivity and specificity.

With an area under the ROC curve of 0.705, the prediction of mortality by chest CT was found to be a statistically informative test with *p* = 0.017. Prediction of mortality at the threshold of 30% lung parenchyma lesions had a sensitivity of 0.583 and a specificity of 0.720 (Figure 2).

### 3.4. Chest CT Images Associated with Death

Among the 200 hospitalized patients, only 2 had been transferred to other health structures. Of the remaining 198 patients, 12 cases of death were recorded (6.1%): 9 deaths (8.2%) in G1 and 3 deaths (3.4%) in G2 (*p* = 0.162). The multivariate analysis retained the extent of lesions > 50% as factor independently associated with death (Table 3).

## 4. Discussion

The interest of this study was to evaluate the contribution of early chest CT in the description of pulmonary lesions and the determination of the prognosis in patients hospitalized in the Congolese environment for suspected COVID-19 and to compare the results with the data of literature.

Similar to observations worldwide, this study showed that ground glass, crazy paving, and consolidation lesions were characteristic of COVID-19 pneumonia, thus confirming the value of chest CT for diagnostic purposes [6–10]. Despite the nonspecific results of lesions that can also be seen in other viral infections, in times of pandemic, this information is an effective and precise tool for first-line triage when the virological diagnosis is delayed or proves doubtful.

We did not evaluate its specificity and sensitivity because it would have been necessary to compare it with the gold standard, “viral culture,” which is not carried out routinely [16]. RT-PCR for the research for viral RNA could not be an alternative to viral culture because; although it is the reference examination for the diagnosis of SARS-CoV-2 infection, it provides itself many false positive or negative results [17]. However, studies that evaluated chest CT in relation to RT-PCR report a sensitivity generally greater than 90% and a specificity varying between 25% and 70% [18, 19].

A temporality is described in the appearance of the lesions on the CT: we initially noted ground-glass opacities, then crazy paving, and finally consolidations [6, 7, 9]. Unfortunately, we did not observe this pattern as the chest CT were not repeated in our series. However, we observed an association of these 3 lesions in most of the patients. This implies that the patients were referred lately to the hospital and were admitted at the stage of condensation lesions.

In the literature, other radiologic signs were reported such as the presence of fine crosslinks, peribronchovascular thickening, and peri- or intralesional vascular dilatation. There were generally no associated excavations, septal lines, or mediastinal adenomegaly in SARS-CoV-2 pneumonia [6–9]. These observations were also confirmed in our study. In 9 patients, atypical pseudonodular lesions were noted, as described by certain authors [20].

Of different signs of severity reported in the literature (extent of lesions, parenchymal condensations, associated pleural effusion, early architectural distortion with traction bronchiectasis, and initial involvement of the upper lobes) [9, 10, 21], only the extent of lesions was retained in the present study. Beyond 50% damage to the lung parenchyma, the probability of death was multiplied by 7 despite optimal management in accordance with the national protocol for the management of COVID-19 patients. Francone et al. had shown that CT scoring could help stratify patient risk and predict the short-term outcome of patients with COVID-19 pneumonia [22]. Furthermore, the extent of lung lesions is strongly correlated with various disease parameters, including clinical stage and laboratory parameters [22, 23].

Cases of pulmonary embolism were not observed in our study. This is also the case in several studies published by African authors [11–14]. In our situation, this can be explained by the fact that these chest CT from the first day of hospital admission were not repeated. There is also the fact that the study was carried out at a time when anticoagulants were systematically prescribed for prevention in moderate and severe forms of COVID-19 in the DR Congo. Fibrosis lesions are described at very late stages of the disease [9, 22]. Definitive conclusions cannot be withdrawn in this study because the chest CT scans were only performed once at admission.

Among the known clinical risk factors for death, hypertension was retained in the multivariate analyses. Older age and diabetes were not retained probably due to the small sample size. However, the different clinical and biological risk factors for mortality in COVID-19 patients have already been identified in several studies in our environment [14, 24, 25]. Our study focused more on the search for chest CT risk factors for mortality.

Among the weaknesses of this study, we note that it was conducted in a single hospital and the sample size was small. Another weakness is that it is a retrospective study. However, this study has the advantage of having analyzed the data from the chest CT scan taken on the day of admission. The assessment of lesions was not visual but used digital software, and the study demonstrated the benefit of early chest CT to assess the prognosis of COVID-19 patients.

## 5. Conclusion

The early chest CT demonstrated certain characteristic lesions more frequently in patients in whom the diagnosis of COVID-19 was confirmed. The extent of lesions affecting at least half of the lung parenchyma from the first day of admission to hospital increased the risk of death by a factor of 7. We can assume that early chest CT can help predict the vital outcome of patients with COVID-19 pneumonia.

## Figures and Tables

**Table 1:** General characteristics of patients.

	Total	SARS-CoV-2 RT-PCR		*p*
	*n* = 200	Positive (*n* = 112)	Negative (*n* = 88)	
Men	114 (57.0)	66 (58.9)	48 (54.5)	0.534
Age (years)	56.2 ± 15.8	56.7 ± 15.1	55.5 ± 16.6	0.582
<40 years	36 (18.0)	19 (17.0)	17 (19.3)	0.801
40 to 59 years old	73 (36.5)	43 (38.4)	30 (34.1)	—
≥60 years old	91 (45.5)	50 (44.6)	41 (46.6)	—
Diabetes	38 (19.0)	22 (19.6)	16 (18.2)	0.794
Hypertension	69 (34.5)	58 (51.8)	11 (12.5)	0.001
Obesity	9 (4.5)	3 (2.7)	6 (6.8)	0.161

Results are presented as absolute value (percentage) or as mean ± standard deviation.

**Table 2:** Chest CT lesions in patients.

	Total	SARS-CoV-2 RT-PCR		*p*
	*n* = 200	Positive (*n* = 112)	Negative (*n* = 88)	
Ground glass	116 (58)	81 (72.3)	35 (39.8)	<0.001
Crazy paving	93 (46.5)	78 (69.6)	15 (17.0)	<0.001
Consolidation	114 (57)	94 (83.9)	20 (22.7)	<0.001
Bilateral lesions	104 (52)	77 (68.8)	27 (30.7)	<0.001
Lesions < 25%	139 (69.5)	64 (57.1)	75 (85.2)	<0.001
25–50%	33 (16.5)	27(24.1)	6 (6.8)	
>50	28 (14)	*n* (18.8)	7 (8.0)	

The results are presented in absolute value (percentage).

**Table 3:** Determinants of death (logistic regression).

Variables	Univariate analysis			Multivariate analysis		
	*p*	OR	95% CI	*p*	OR	95% CI
*Whole group*						
Lesions > 30%	0.033	3.608	1.096–11.876			
Lesions > 50%	0.011	5.612	1.634–19.282	0.006	5.618	1.634–19.231
Male gender	0.023	4.118	0.878–19.301	0.066	4.367	0.901–20.833
Hypertension	0.004	6.414	1.674–24.576	0.005	6.897	1.770–27.027
*COVID-19 subgroup*						
Lesions > 50%	0.007	7.768	1.857–32.486	0.008	7.194	1.656–31.250

Other variables are introduced but not retained in the model: confirmed SARS-CoV-2 infection, bilateral lesions vs. unilateral lesions, type of lesions (consolidation vs. crazy paving vs. ground glass), age > 60 years, and diabetes.

## Data Availability

The data used to support the findings of this study are available from the corresponding author upon request. Data were processed and analyzed anonymously and were kept strictly confidential. All data were fairly treated.
